# Five-year longitudinal surveillance reveals the continual circulation of both alpha- and beta-coronaviruses in Plateau and Gansu pikas (*Ochotona* spp.) at Qinghai Lake, China^1^

**DOI:** 10.1080/22221751.2024.2392693

**Published:** 2024-08-13

**Authors:** Lin Xu, Meiqing Song, Xianzhi Tian, Ju Sun, Yanjun Wang, Mengyu Bie, Yuhai Bi, Edward C. Holmes, Yi Guan, Jianjun Chen, Juan Li, Weifeng Shi

**Affiliations:** a School of Public Health, Shandong First Medical University & Shandong Academy of Medical Sciences, Ji'nan, People’s Republic of China; b Key Laboratory of Emerging Infectious Diseases in Universities of Shandong, Shandong First Medical University & Shandong Academy of Medical Sciences, Ji'nan, People’s Republic of China; c CAS Key Laboratory of Pathogen Microbiology and Immunology, Institute of Microbiology, Center for Influenza Research and Early-warning (CASCIRE), CAS-TWAS Center of Excellence for Emerging Infectious Diseases (CEEID), Chinese Academy of Sciences (CAS), Beijing, People’s Republic of China; d University of Chinese Academy of Sciences, Beijing, People’s Republic of China; e School of Medical Sciences, The University of Sydney, Sydney, Australia; f Ruijin Hospital, Shanghai Jiao Tong University School of Medicine, Shanghai, People’s Republic of China; g Shanghai Institute of Virology, Shanghai Jiao Tong University School of Medicine, Shanghai, People’s Republic of China; h State Key Laboratory of Virology, Wuhan Institute of Virology, Chinese Academy of Sciences, Wuhan, People’s Republic of China

**Keywords:** Alphacoronavirus, betacoronavirus, pika, evolution, Qinghai Lake

## Abstract

The discovery of alphacoronaviruses and betacoronaviruses in plateau pikas (*Ochotona curzoniae*) expanded the host range of mammalian coronavirus (CoV) to a new order – Lagomorpha. However, the diversity and evolutionary relationships of CoVs in these plateau-region-specific animal population remains uncertain. We conducted a five-year longitudinal surveillance of CoVs harboured by pikas around Qinghai Lake, China. CoVs were identified in 33 of 236 plateau pikas and 2 of 6 Gansu pikas (*Ochotona cansus*), with a total positivity rate of 14.5%, and exhibiting a wide spatiotemporal distribution across seven sampling sites and six time points. Through meta-transcriptomic sequencing and RT–PCR, we recovered 16 near-complete viral genome sequences. Phylogenetic analyses classified the viruses as variants of either pika alphacoronaviruses or betacoronaviruses endemic to plateau pikas from the Qinghai-Tibet Plateau region. Of particular note, the pika-associated betacoronaviruses may represent a novel subgenus within the genus *Betacoronavirus*. Tissue tropism, evaluated using quantitative real-time PCR, revealed the presence of CoV in the rectal and/or lung tissues, with the highest viral loads at 10^3.55^ or 10^2.80^ RNA copies/μL. Surface plasmon resonance (SPR) assays indicated that the newly identified betacoronavirus did not bind to human or pika Angiotensin-converting enzyme 2 (ACE2) or Dipeptidyl peptidase 4 (DPP4). The findings highlight the ongoing circulation and broadening host spectrum of CoVs among pikas, emphasizing the necessity for further investigation to evaluate their potential public health risks.

## Introduction

Members of the family *Coronaviridae* are enveloped, positive-sense RNA viruses that are among the largest RNA viruses identified to date. The latest classification by the International Committee on Taxonomy of Viruses (ICTV) recognizes three subfamilies within the *Coronaviridae*: the *Orthocoronavirinae*, *Letovirinae*, and *Pitovirinae* [1]. Coronavirus (CoV) is the collective term for the members of the subfamily *Orthocoronavirinae* that comprise four genera: *Alphacoronavirus*, *Betacoronavirus*, *Gammacoronavirus*, and *Deltacoronavirus*. Among the nine known human-infecting CoVs, Human coronaviruses (HCoV) 229E [2], HCoV-NL63 [3], and canine coronavirus (CCoV) [4], are classified within the genus *Alphacoronavirus*, porcine coronavirus HKU15 (also known as porcine deltacoronavirus) belongs to the genus *Deltacoronavirus* [5], while the remaining five viruses are members of the genus *Betacoronavirus*: HCoV-OC43 [6], HCoV-HKU1 [7], Severe acute respiratory syndrome coronavirus (SARS-CoV) [8], Middle East Respiratory Syndrome coronavirus (MERS-CoV) [9], and SARS-CoV-2 [10, 11]. Of the human viruses, HCoV-229E, HCoV-NL63, HCoV-OC43, and HCoV-HKU1 typically cause mild respiratory infections and common colds, while SARS-CoV, MERS-CoV, and SARS-CoV-2 are capable of causing epidemics and even pandemics of systemic infection in humans [1].

CoVs infect a wide host range of animals, including birds and mammals (bat, rodent, canine, feline, cattle, and swine, etc.) [1]. The precise identification of CoV hosts is of major importance in blocking viral spread from animal sources, preventing prolonged transmission, and averting disease recurrence. SARS-CoV and MERS-CoV have been unequivocally linked to wildlife origins, with bats serving as the natural hosts [12–14]. While the proximal source of SARS-CoV-2 remains elusive, the most probable transmission route involves a long-term reservoir in *Rhinolophus* bats with an as yet unidentified intermediate host facilitating zoonotic transfer to humans [15]. Although considerable attention has been directed to bats as hosts for CoVs, rodents have been proposed as the original hosts of HCoV-OC43, yet substantive evidence is still lacking [16, 17]. The natural hosts for HCoV-229E, HCoV-NL63, and HCoV-HKU1 remain elusive [18, 19]. In February 2024, the World Health Organization issued a cautionary statement: “History teaches us that the next pandemic is a matter of when, not if. It may be caused by an influenza virus, or a new coronavirus, or it may be caused by a new pathogen we don’t even know about yet – what we call Disease X.” This underscores the ongoing need to monitor zoonotic CoV reservoirs and the risk of cross-species transmission.

Pikas belong to the family Ochotonidae (order Lagomorpha) and consist of a single extant genus, *Ochotona*, with 30 currently recognized species [20]. Recent studies have detected various viruses, including Influenza virus H5N1 [21], H9N2 [22], H7N2 [23], arenavirus (known as plateau pika virus, PPV) [24], and mammalian orthoreovirus (MRV) [25], in the plateau pikas (*Ochotona curzoniae*), the most populous mammal on the Qinghai-Tibet Plateau [26]. These viruses cause infection *in vivo* [27]. As such, it is essential to explore the potential zoonotic viruses carried by plateau pikas to assess the biosafety risks in this unique habitat. Indeed, a survey of pathogens within animal populations in the Qinghai-Tibet Plateau region uncovered a new alphacoronavirus in plateau pikas [28], while novel betacoronaviruses were identified in plateau pikas at the edge of the plateau in Sichuan province, China [29]. These findings underscore that pikas harbour various CoVs, with more yet to be identified, and highlight the necessity for a comprehensive investigation of the genetic diversity of pika-borne CoVs and their potential for zoonotic transmission to humans.

In this study we conducted a comprehensive investigation of CoVs in pikas inhabiting the area surrounding Qinghai Lake, where pikas are at high population size. Alphacoronaviruses and betacoronaviruses were detected in two pika species, harbouring substantial genetic diversity and virus circulation between sampling times and locations. In addition, we examined the receptor-binding characteristics of pika betacoronaviruses with respect to Angiotensin-converting enzyme 2 (ACE2) and Dipeptidyl peptidase 4 (DPP4, also known as CD26).

## Materials and methods

### Ethics statement

The procedures for sampling were reviewed and approved by the ethical committee of the Shandong First Medical University & Shandong Academy of Medical Sciences (No. 2019033). All institutional and national guidelines for the care and use of animals were followed.

### Sample collection

**Figure 1.**
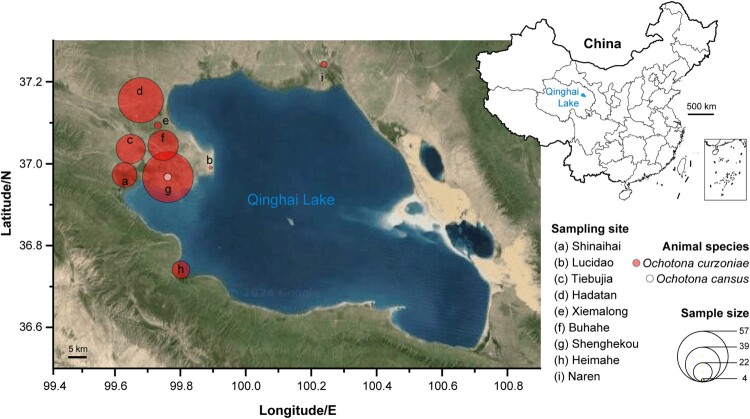
Pika sampling around Qinghai Lake, Qinghai province, China. Map of Qinghai Lake showing nine sampling sites with accurate coordinates. Circles on the map show the geographical sites where the pikas were surveyed. The circle colours indicate the animal species according to the legend.

To survey pika-borne pathogens, field expeditions were conducted spanning ten time points between 2019 and 2023 at nine sampling sites surrounding Qinghai Lake in China (Qinghai province). This resulted in the collection of 242 pikas (Table 1). The sampling sites are marked as a to i in Figure 1. Pika species were morphologically identified by an experienced expert and confirmed by sequencing of the mitochondrial cytochrome *b* gene (cyt *b*) [30]. In total we sampled 236 plateau pikas and six Gansu pikas (*Ochotona cansus*). The plateau pikas were collected at all nine sampling locations with the numbers of captured animal ranging from four (Lucidao, b) to 52 (Shenghekou, g), whereas Gansu pikas were only sampled at two sites, Shinaihai (*n *= 1) and Shenghekou (*n *= 5). Animal dissection was performed individually under ether (Sigma-Aldrich, USA) anaesthesia, and heart, liver, spleen, lung, kidney, rectum, and brain tissues were collected.

**Table 1.** Information on pika sample collection and CoV detection.

Sampling site	Sampling date										Total
	Jul 2019	Aug 2020	May 2021	Jun 2021	Jul 2021	Jun 2022	Jul 2022	May 2023	Jun 2023	Jul 2023	
Shinaihai (a)^a^	0/24							0/3			0/28
	0/1										0/1
Lucidao (b)	1/4 (25.0)										1/4 (25.0)
Tiebujia (c)			0/16	2/17 (11.8)							2/33 (6.1)
Hadatan (d)		0/19				1/6 (16.7)	1/2 (50.0)	0/2	3/17 (17.6)	1/5 (20.0)	6/51 (11.8)
Xiemalong (e)			0/8								0/8
Buhahe (f)				0/10	0/17		1/7 (14.3)				1/34 (2.9)
Shenghekou (g)^a^			0/2			11/16 (68.8)	2/3 (66.7)	0/6	0/11	3/14 (21.4)	16/52 (30.8)
						1/1 (100)	1/4 (25.0)				2/5 (40.0)
Heimahe (h)						0/9	1/6 (16.7)			0/5	1/20 (5.0)
Naren (i)									4/4 (100)	2/3 (66.7)	6/7 (85.7)
Total	1/29 (3.4)	0/19	0/26	2/27 (7.4)	0/17	14/32 (40.6)	6/22 (27.3)	0/11	8/32 (21.9)	6/27 (22.2)	35/242 (14.5)

Note: The data shows CoV-positive/total No. of pikas by RT-nPCR. The numbers in parentheses indicate the percentage that are CoV positive.

^a^ For the Shinaihai and Shenghekou sampling sites, numbers in the top and bottom rows show the data for the plateau pikas and Gansu pikas, respectively. In the case of the remaining sites, all of the numbers represent plateau pikas.

### Cov screening by reverse transcription nested PCR (RT-nPCR)

To screen potential CoVs in pikas, RT-nPCR was performed by targeting a 440-bp gene region of the RNA-dependent RNA polymerase (RdRp) gene using the pan-CoV degenerated primers (Table S1) [31]. Approximately 25 mg of each tissue was homogenized in 500 μL of sterile phosphate-buffered saline (PBS) solution (Gibco, USA), and was centrifuged at 8000×*g* for 8 min. For each animal tested, equivalent supernatants of different types of tissue were pooled, and RNA was extracted using the RNeasy Plus Mini Kit (Qiagen, Germany). Complementary DNA (cDNA) was obtained using the PrimeScript 1st Strand cDNA Synthesis Kit (TaKaRa, Japan), and was used as the template for CoV screening employing the 2×Taq PCR Mix (Tiangen, China) with the following PCR programs: initial denaturation at 94°C for 30 s, followed by 30 cycles (outer PCR) or 35 cycles (inner PCR) of denaturation at 94°C for 30 s, annealing at 54°C for 30 s, and extending at 72°C for 40 s, with double-distilled water (ddH_2_O) as a negative control. Amplicons were then characterized by Sanger sequencing (Sangon Biotech, China).

To confirm the presence of CoVs in the original tissue samples, RNA was extracted separately from the heart, liver, spleen, lung, kidney, rectum and brain tissues of the positive individuals, with RT-nPCR and Sanger sequencing then performed as described above.

### Meta-transcriptomic sequencing

To obtain the genome sequences of the CoVs identified, the RNA extracted from the positive pooled samples were sequenced using a meta-transcriptomic sequencing protocol. RNA integrity and quantity were measured using the RNA Nano 6000 Assay Kit of the Bioanalyzer 2100 system (Agilent Technologies, USA). For all libraries, we followed the TruSeq stranded total RNA paired-end libraries protocol (Illumina, USA). Briefly, following DNase I digestion, the ribosomal RNA (rRNA) was depleted from total RNA using the Ribo-Zero Plus Kit (Illumina, USA) following the manufacturer’s instructions. The remaining RNA was fragmented, reverse-transcribed, ends adenylated and adaptor-ligated. After purification of cDNA, amplification was performed by polymerase chain reaction (PCR). Paired-end (150 bp) sequencing of each RNA library was performed on the NovaSeq 6000 platform (Illumina, USA). All library preparation and sequencing were performed by Novogene Company (Beijing, China).

### Transcriptome analysis and genome annotation

Raw sequencing reads were quality-controlled and preprocessed with the Fastp programme v0.20.0 [32]. Eukaryotic rRNAs were removed with Bowtie2 v2.3.3.1 [33], and the resultant reads were *de novo* assembled using Trinity v2.5.1 employing default settings [34]. The assembled contigs were compared with the non-redundant nucleotide (nt) and protein (nr) database downloaded from GenBank using Blastn with an e-value cutoff at 1E-1 and Diamond Blast with an e-value cutoff at 1E-5 [35]. All contigs were filtered to remove the host, plant, bacterial, and fungal sequences. For CoV in an individual library, contigs were further merged using Geneious Prime v2023.2.1 (Biomatters, New Zealand).

Primers with between 100 and 300 bp overlapping regions were designed to fill gaps in the CoV genome sequences (Table S1), and PCR was performed to generate amplicon. These amplicons were then bidirectionally sequenced using Sanger sequencing, and the fragments obtained were assembled to recover the complete genome sequences. Potential open reading frames (ORFs) were predicted using ORFfinder (https://www.ncbi.nlm.nih.gov/orffinder/), and the conserved protein domains were annotated using the Conserved Domain Search (https://www.ncbi.nlm.nih.gov/cdd).

### Calculation of virus abundance

The relative abundance of the CoVs in the libraries was determined by mapping the reads back to the assembled contigs using Bowtie2 v2.3.3.1 and then calculating the number of reads per million (RPM): that is, the number of reads mapped to the CoV contigs divided by the total number of reads in a library after the removal of rRNA reads and multiplied by a million [33]. A CoV is considered “abundant” in a library if its reads accounted for more than 0.1% of the non-rRNA reads [36].

### Phylogenetic analyses

To determine the phylogenetic relationships among the newly identified and previously described CoVs, reference sequences were downloaded from GenBank and were aligned using MAFFT v7.450 [37]. Phylogenetic trees were then estimated using the maximum likelihood (ML) method in PhyML v3.0 employing 1000 bootstrap replicates to determine node support [38]. As proposed by the ICTV, the subgenus and species demarcation thresholds of CoVs were set as 0.132-0.142 and 0.075 pairwise uncorrected distances (PUDs), respectively. These distances were calculated based on a multiple amino acid alignment of 3C-like protease (3CLpro, nsp5), RdRp (nsp12), and Zinc-binding domain-containing Helicase (HELI, nsp13) [39, 40]. Pairwise and between-group mean distances were calculated using MEGA 7.0, employing a bootstrap value of 1,000 [41]. Similarity plots were inferred by using Simplot v3.5.1 to characterize potential recombination events between the newly identified members and other CoVs with a window size of 200 bp and a step size of 20 bp [42].

### Development of the quantitative real-time PCR (qPCR) assays

To measure the viral load of the CoVs in the positive samples, alphacoronavirus- or betacoronavirus-specific qPCR detection assays were established. The partial RdRp sequence (∼440 bp) of the newly discovered CoVs amplified by nPCR was purified and cloned into the pMD19-T vector (TaKaRa, China), and was used as standard plasmid and positive control. Specific oligonucleotide primers and a probe labelled with 5′-FAM and 3′-BHQ1 targeting the RdRp gene were designed and synthesized (Table S1). qPCR was performed using the 2× Pro Taq HS Premix Probe qPCR Kit III (Accurate Biology, China). To determine the optimal reaction composition and thermocycling conditions, preliminary experiments with different primer and probe concentrations, as well as annealing temperature, were conducted. Finally, the qPCR reaction was performed in a 20 µL reaction system that contained 2 µL of cDNA template, 0.4 µM of specific oligonucleotide forward and reverse PCR primers and probe, and 10 µL of 2× Pro Taq HS Probe Premix (Accurate Biology, China). The qPCR was performed with the following procedures: 95°C for 10 min, then 45 cycles of denaturation at 95°C for 15 s and annealing at 60°C for 30 s. The fluorescence signal was automatically collected at the end of each cycle. Three replicate wells were allocated for each sample with RNase-free water used as negative control. The cycle threshold (*Ct*) values were generated with ABI QuantStudio 1 Plus instrument (Thermo, USA), and the results were analysed using the QuantStudio Design & Analysis SC Software v1.0. Viral loads were calculated as log_10_ RNA copies/μL based on the *Ct* values of the samples and the standard curve. Mean values from the triplicates were displayed with error bars indicating standard deviation (SD).

### Viral particle observation

To examine whole CoV particles, tissues with high genome copy numbers were homogenized with Minimum Essential Medium (MEM, Gibco, USA) and centrifuged 12,000×*g* for 15 min at 4°C to remove cell debris. The resultant supernatant was then filtered using a 0.22 μm sterile filter (Millipore, USA), and used for observation by transmission electron microscopy (TEM) at 80 kV (Hitachi, Japan) after negative staining with 5% phosphotungstic acid as previously described [43].

### Protein expression and purification

The coding sequences of the receptor-binding domain (RBD) of pika betacoronavirus strain NQ06 (spike residues 313-623), SARS-CoV-2 strain hCoV-19/Wuhan/IVDC-HB-01/2019 (spike residues 319-541, GISAID: EPI_ISL_402119) and MERS-CoV strain HCoV-EMC/2012 (spike residues 368-586, GenBank Accession No.: NC_019843) with a 6× His Tag were synthesized after codon optimization, and cloned into the pCAGGS vector. Pika-ACE2 (residues 1-740, NC_080865), pika-DPP4 (residues 30-765, NC_080836), hACE2 (residues 1-740, AY623811), and hDPP4 (residues 39-766, NM_001935) with the IgK signal peptide and a 6× His Tag were also synthesized after codon optimization and cloned into the pCAGGS vector. All of the recombinant plasmids were transiently transfected into Expi293F cells. After five days, the culture supernatants were collected and the soluble protein was purified using a 5 mL His-Trap HP column (GE Healthcare, USA) in PBS buffer (1.8 mM KH_2_PO_4_, 10 mM Na_2_HPO_4_, 137 mM NaCl, 2.7 mM KCl, pH 7.4). The eluted protein was further purified using a Superdex 200 Increase 10/300 GL column (GE Healthcare, USA) in a PBST buffer (1.8 mM KH_2_PO_4_, 10 mM Na_2_HPO_4_, 137 mM NaCl, 2.7 mM KCl, and 0.005% (v/v) Tween 20 pH 7.4).

### Surface plasmon resonance (SPR) assay

The affinities and kinetics of the RBDs binding to pika and human receptor analogues were analysed using BIAcore 3000 system at 25°C with CM5 chips (GE Healthcare, USA). Briefly, the RBD proteins of NQ06, SARS-CoV-2, and MERS-CoV were immobilized on the chip at approximately 1000 response units (RUs), and PBST was set as the negative control. The receptor proteins, pika-ACE2, pika-DPP4, hACE2, or hDPP4 were prepared and used to flow over the chip surface, respectively. Association of the receptor analytes (1, 10, and 100 μM) was measured at a flow rate of 30 μL/min. After each cycle, the sensor surface was regenerated via a short treatment using 10 mM NaOH. Binding kinetics were analysed using a 1:1 Langmuir binding and/or steady-state affinity models using BIAevaluation version 4.1 software (GE Healthcare, USA).

## Results

### Prevalence of alphacoronaviruses and betacoronaviruses in pikas

Among the 242 pikas tested, 35 were positive for CoVs by RT-nPCR, yielding a total positivity rate of 14.5%. These included 33 plateau pikas, with a species-specific positivity rate of 14.0%, and two Gansu pikas with a positivity rate of 33.3%. The CoVs showed a wide spatiotemporal distribution in pikas: six of the ten sampling time points across July 2019 to July 2023 contained positive samples, with positivity rates ranging from 3.4% (1/29) in July 2019 to 40.6% (14/32) in June 2022. Similarly, seven of the nine sampling sites contained positive samples, with positivity rates ranging from 2.9% (1/34) at Buhahe (f) to 85.7% (6/7) at Naren (i) (Table 1). Of particular note, the 52 plateau pikas and five Gansu pikas collected at Shenghekou showed a high CoV-positivity rate of 30.8% and 40.0%, respectively.

Partial RdRp sequences (∼400 bp) of the 35 strains obtained from the primer removal of 440-bp sequences by RT-nPCR were used for preliminary phylogenetic analysis (Figure S1) and CoV annotation (Table S2). Six sequences belonged to the genus *Alphacoronavirus*, with nucleotide identities of 99.7–100% among themselves. They were detected at Shenghekou (g) in July 2022 (*n *= 3) and July 2023 (*n *= 3), and showed the closest relationship (98.2–98.5% nucleotide identity) to Plateau pika coronavirus P83 (GenBank accession No.: MZ577265) which was also identified from plateau pikas in Yushu Tibetan Autonomous Prefecture in Qinghai province in 2019 [28]. The other 29 strains were found at seven sampling sites across six time points, and held identities of 98.0–100% among themselves, and all belonged to the genus *Betacoronavirus*. They exhibited the closest relationship (97.0–98.2% nucleotide identity) with Pika coronavirus SC/C3-17.18/2021 (OQ297696) that was detected in Gansu pika in the Qinghai-Tibet plateau region of Sichuan province in 2021 [29]. The names of the newly identified alphacoronaviruses and betacoronaviruses were then designated according to their host species, sampling time, and sites. For example, the betacoronavirus found in plateau pika #NQ06 was named as Pika betacoronavirus strains OCu-NQ06/Qinghai/2022 (NQ06 for short).

### Genome sequencing by meta-transcriptomics

The 35 positive pooled samples were then subjected to meta-transcriptomic sequencing. This generated 336.63 GB of data comprising ∼2.15 billion raw reads. Detailed information on the meta-transcriptomic sequencing is summarized in Table S3. After adapter trimming and quality control, 312.69 GB of clean data were obtained, of which 118.79 million reads remained after the removal of rRNA. After *de novo* assembly and comparison with the non-redundant nt and nr databases, a total of ∼224.2 thousand reads were annotated to CoVs, accounting for 0.15% of the total non-rRNA reads. Within each library, the relative abundance values of CoV reads were calculated as RPM that ranged from 1.14 (NQ34) to 20,717.30 (NQ39). Five libraries (NQ06, NQ32, NQ39, NRSD01, and SHK13) in which the reads of CoVs accounts for more than 0.1% of the non-rRNA reads were regarded CoV abundant.

### Genome organization

**Figure 2.**
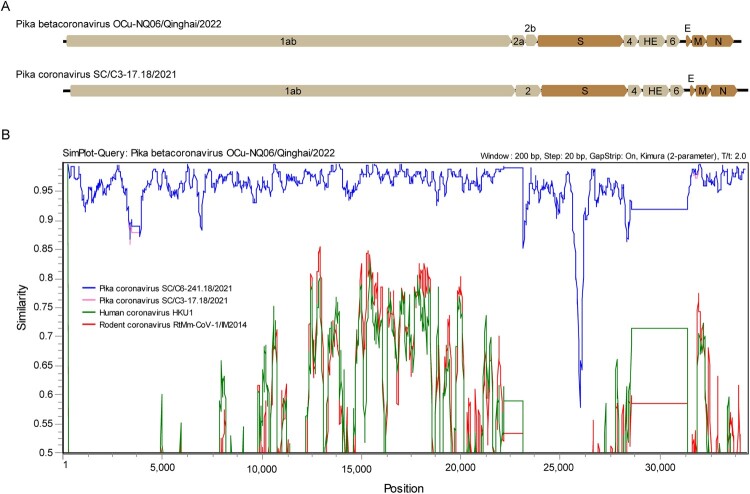
Predicted genome organization and whole-genome similarity between the newly identified betacoronavirus and close reference sequences.

After gaps were filled with overlapped PCR, 16 near-complete genome sequences (with partial 5′- and 3′-termini) were obtained, including two sequences of alphacoronaviruses and 14 sequences of betacoronaviruses. The two alphacoronaviruses, NQ37 and NQ39, had genome sizes of 28,297 bp and 28,299 bp, respectively, both with G+C contents of 35.9%. The predicted genome organizations of NQ37 and NQ39 showed the same gene order as Plateau pika coronavirus P83: 5′-1ab-2-S-4-E-M-7a-N-9-3′ (Table S4). The genome sizes of the 14 betacoronaviruses ranged from 32,096 bp to 32,285 bp, with G+C contents of 35.9–36.0%. They possessed the same genome organization, and were similar with Pika coronavirus SC/C3-17.18/2021, with the exception of the predicted ORF2a (702 bp) and ORF2b (558 bp) instead of ORF2 (1,230 bp) as present in the reference sequence. This resulted in the following order of proteins: 5′-1ab-2a-2b-S-4-HE-6-E-M-N-3′ (Figure 2(A) and Table S4).

### Phylogenetic relationships

**Figure 3.**
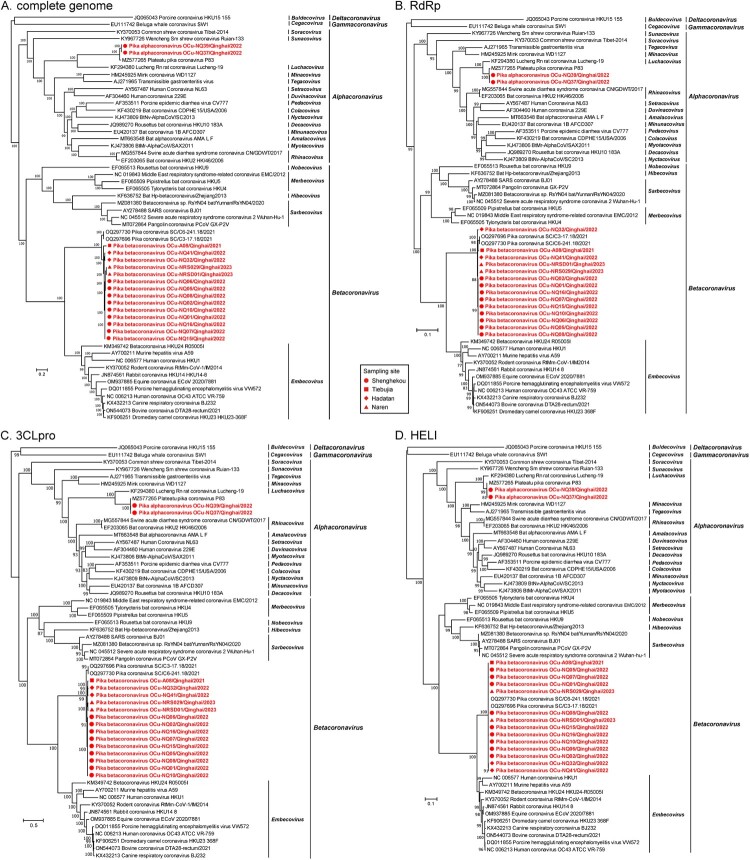
Phylogenetic relationships of the CoVs identified in this study and representatives from the family *Coronaviridae*. ML phylogenetic trees were generated based on (A) the complete genomic sequences, the complete amino acid sequences of (B) RdRp, (C) 3CLpro, and (D) HELI proteins. The sequences identified here are marked in red, while the sampling sites are labelled according to the legend. The viral subgenus and genus names are shown in italics. The tree is rooted on the *Deltacoronavirus* and *Gammacoronavirus* outgroups, with branch lengths scaled according to the number of substitutions per site. Bootstrap values are shown at the tree nodes.

Phylogenetic analysis of the complete genome sequences showed that the 16 CoV sequences identified here formed two clusters. The alphacoronaviruses strains NQ37 and NQ39 had identical genome sequences and clustered with Plateau pika coronavirus P83 in the genus *Alphacoronavirus* with a nucleotide identity of 97.8% (Figure 3(A) and Table S5). In turn, these sequences were related to Lucheng Rn rat coronavirus Lucheng-19 (KF294380) from the subgenus *Luchacovirus* [44]. In a similar manner, the betacoronavirus sequences shared nucleotide identities of 96.1–100%, exhibiting nucleotide variations throughout the genomes, particularly in the S-4-HE region (Figure S2). Of note, the betacoronavirus sequences varied across time points. Specifically, nine of the 14 strains detected in June 2022 exhibited high sequence identities of 99.9–100% with each other, and 98.8–98.9% and 98.5% with the two strains (NQ32 and NQ41) sampled in July 2022 and the two strains (NRSD01 and NRS029) sampled in 2023, respectively. In addition, viral strain A08, detected in the 2021 samples, displayed a relatively distant relationship to other strains, with sequence identities of 96.1–96.9%. In the phylogenetic tree, the betacoronaviruses clustered with Pika coronavirus SC/C3-17.18/2021 and its sister strain (Pika coronavirus SC/C6-241.18/2021) with nucleotide identities of 94.5–95.9% (Table S5).

Comparison of the amino acid sequences of the RdRP, 3CLpro, and HELI also showed that the pika alphacoronaviruses shared the highest identities with Plateau pika coronavirus P83 (99.6%, 98.7%, and 99.8%, respectively) and exhibited the same topological position in phylogenetic analyses, whereas the pika betacoronaviruses were most similar to Pika coronavirus SC/C3-17.18/2021 (98.8–99.2%, 94.5–95.8%, and 100%, respectively), although their relative positions varied between phylogenies (Figure 3(B–D)). With respect to amino acid similarity, the pika alphacoronaviruses identified in this study exhibited sequence distances of 0.013 (3CLpro), 0.004 (RdRp), and 0.002 (HELI) with Plateau pika coronavirus P83, and 0.066, 0.396, and 0.064 with Lucheng Rn rat coronavirus Lucheng-19 [44] (Table S6). These values were all below the species demarcation threshold of 0.075 proposed by the ICTV [1, 39, 40], indicating that they represented novel variants of Lucheng-19 within the subgenus *Luchacovirus*. The newly discovered pika betacoronaviruses showed amino acid sequence distances of 0.041–0.046 (3CLpro), 0.008–0.012 (RdRp), and 0 (HELI) with Pika coronavirus SC/C3-17.18/2021, revealing them as novel variants of this virus (Table S6). Also, of note was that the between-group mean amino acid distances to the subgenus *Embecovirus* were 0.206–0.230, 0.558–0.589, and 0.104–0.164 in the three proteins, respectively. Considering the subgenus demarcation thresholds (0.132–0.142) [1, 39, 40], these values suggest that the newly found pika betacoronaviruses along with the references may belong to a novel subgenus.

Comparison of the spike protein revealed high amino acid identity of 94.0% between the pika alphacoronaviruses identified in this study and Plateau pika coronavirus P83. The spike protein of the 14 betacoronaviruses possessed sequence identities of 87.4–100% with each other, and 87.0–100% to Pika coronavirus SC/C3-17.18/2021. The furin cleavage site (RRAR) present between S1 and S2 identified in SARS-CoV-2 was absent in the deduced spike protein of pika betacoronaviruses (Figure S3). Phylogenetic analysis of the spike protein sequences also revealed similar tree topologies to those described above; however, strain A08 was placed at the root of the pika betacoronavirus clade (Figure S4).

To characterize potential recombination events, sequence similarity plots were inferred between the newly identified CoVs and other members of the *Alphacoronavirus* or *Betacoronavirus* genera. No evidence for recombination was found in the strains described here (Figure 2(B)).

### Viral load quantification in tissues

**Figure 4.**
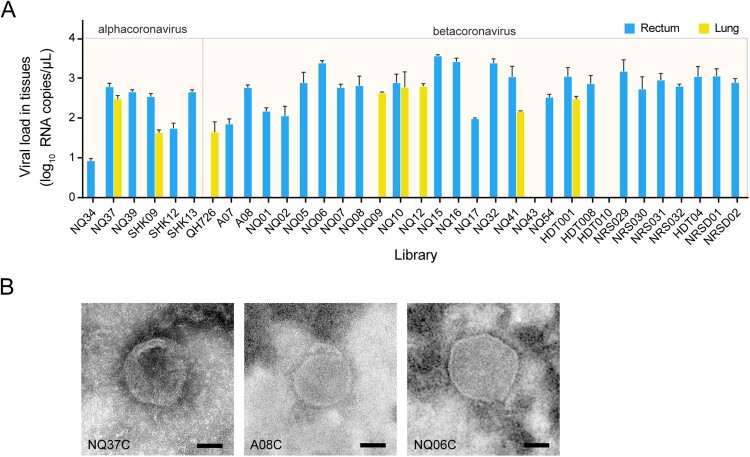
CoV characterization in the pika tissues by qPCR and TEM. (A) RNA loads in tissue samples were assayed by RT-qPCR. Mean values from triplicates are shown with error bars indicated by SD. (B) Viral particles observed in the rectum samples of alphacoronavirus NQ37 (NQ37C), and betacoronaviruses A08 (A08C) and NQ06 (NQ06C) using TEM at a magnification of 80,000 times. The scale bar represents 50 nm.

Tissue specimens involved in the positive pooled samples were assayed using the alphacoronavirus- or betacoronavirus-specific qPCR methods established here. Of the six alphacoronavirus-positive pikas (by nPCR), specific amplification curves were observed in all of the six rectum tissues with the viral loads of 10^0.93^–10^2.78^ RNA copies/μL, and in lung tissues of NQ37 (10^2.48^ RNA copies/μL) and SHK09 (10^1.63^ RNA copies/μL) (Figure 4(A)). In the case of the 29 betacoronavirus-positive pikas, CoV was detected in 24 rectum tissues with the viral loads of 10^1.85^–10^3.55^ RNA copies/μL, and in six lung tissues of 10^1.65^–10^2.80^ RNA copies/μL, among which three pikas, NQ10, NQ41, and HDT001, were positive for both tissues (Figure 4(A)). However, all the other tissue samples including heart, liver, spleen, and kidney tested negative. The viral loads were then calculated as log_10_ RNA copies/μL, ranging between 0.93–3.55 in rectum samples, and 1.63–2.80 in lung tissues (Figure 4(A)).

### Virus morphology characterization

Three original rectum samples of pikas, NQ37C (positive for pika alphacoronavirus), NQ06C and A08C (both positive for pika betacoronaviruses) were used for viral particle examination by TEM. Spherical virions approximately 100–120 nm in diameter were observed in sights of all these samples (Figure 4(B)).

### Receptor-binding properties of pika betacoronaviruses

**Figure 5.**
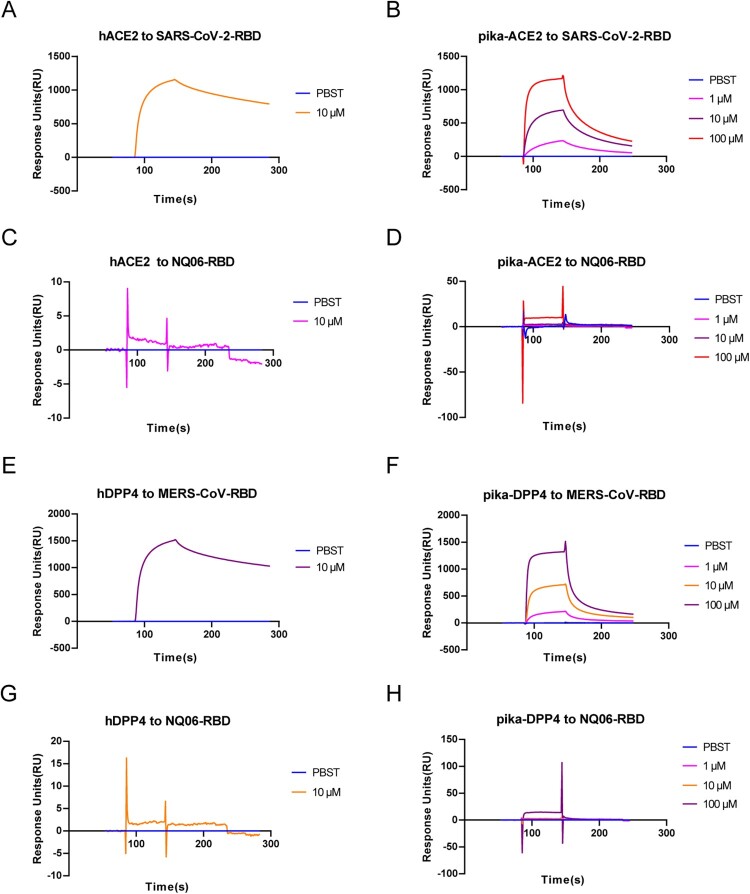
The binding kinetic curves of the RBD of pika betacoronavirus NQ06 with ACE2 or DPP4 plotted by SPR assay. Receptor-binding profiles of RBD of NQ06 with human or pika ACE2 (C and D) or DPP4 (G and H) were assayed at different concentrations, with those of SARS-CoV-2 (A and B) and MERS-CoV (E and F) used as controls.

The binding activities of the newly identified pika betacoronavirus to hACE2 and hDPP4 were characterized by preparing the RBDs of NQ06 using SARS-CoV-2 and MERS-CoV as controls. The SPR experiments demonstrated that SARS-CoV-2-RBD bound to hACE2 as expected (Figure 5(A)), and it was also able to bind to pika-ACE2 (Figure 5(B)) with high affinity. Similarly, MERS-CoV-RBD exhibited high-affinity binding to both to hDPP4 (Figure 5(E)) and pika-DPP4 (Figure 5(F)). However, NQ06-RBD displayed no detectable binding to any of the receptor proteins, including hACE2 (Figure 5(C)), pika-ACE2 (Figure 5(D)), hDPP4 (Figure 5(G)), or pika-DPP4 (Figure 5(H)).

## Discussion

Pikas are native inhabitants of the Qinghai-Tibet Plateau ecosystem, and have frequent interactions with local residents and tourists. They also serve as the primary food source for raptors and carnivorous mammals in the plateau. The plateau pika (*Ochotona curzoniae*) is a key species with a dense population in the Qinghai-Tibet Plateau region, which covers the Tibet Autonomous Region, much of Qinghai province, and extends into western Sichuan province and the southern Uygur Autonomous Region of Xinjiang province [26]. They have been found to carry various viruses, including influenza viruses H5N1 [21], H9N2 [22], and H7N2 [23], arenavirus (plateau pika virus, PPV) [24], and mammalian orthoreovirus (MRV) [25] with *in vivo* infection [27], highlighting the potential risk for cross-species transmission. Alphacoronaviruses and betacoronaviruses have been discovered in the plateau pikas [28, 29], and have also been identified in other wild mammals from the Qinghai-Tibetan Plateau region, including rodents and shrews [45, 46]. However, the role of different pika species in the evolution and transmission of CoVs in the unique plateau habitat remains obscure.

We performed a five-year longitudinal sampling of pikas around Qinghai Lake in China. Alphacoronaviruses and betacoronaviruses were identified in 14.5% of the 242 pikas, including both Gansu and plateau pikas, further expanding the host range of CoVs to a new host species (*Ochotona cansus*). Notably, CoVs were found at seven sampling sites and six sampling occasions: alphacoronaviruses were found during 2022–2023, whereas the newly described betacoronaviruses were identified from 2019 to 2023. These results highlight the continually expanding host range, high prevalence, and constant circulation of CoVs in pika populations.

Phylogenetic analyses revealed that the pika CoVs identified here grouped closely with the alphacoronaviruses and betacoronaviruses previously identified in plateau pikas collected in the Qinghai-Tibet Plateau region [28, 29]. According to the ICTV classification rules for CoVs [1, 39, 40], the alphacoronaviruses described here and Plateau pika coronavirus P83 might represent various variants of Lucheng Rn rat coronavirus Lucheng-19 in the subgenus *Luchacovirus* [44], whereas the pika betacoronaviruses might comprise a new subgenus within the genus *Betacoronavirus*. The newly identified betacoronaviruses exhibited sequence variation across various time points spanning 2021–2023 (Figure S2), thereby demonstrating the ongoing genetic diversity and evolution of betacoronaviruses in pikas.

To elucidate the tissue tropism of the newly identified CoVs, different types of tissue specimen from each CoV-positive pika were tested for CoVs by qPCR. The results showed that the intestines and/or lungs of the pikas were positive, with the highest viral loads of 10^3.55^ and 10^2.80^ RNA copies/μL in the rectum and lung tissues, respectively, whereas other tissues tested including liver, spleen, kidney, and brain were all negative. This was consistent with the screening result of Plateau pika coronavirus P83 [28]. Given that CoV transmission involves multiple routes, such as contact with contaminated surfaces or objects, fomites, respiratory aerosols/droplets, and fecal-oral transmission, the pika population warrants further investigation [47–49].

The receptor-binding profiles are of great importance to understand the host range and zoonotic risk of animal viruses [50–54]. NQ06, a representative strain of the betacoronaviruses described here, was unable to bind with any of the receptors, including pika-ACE2, pika-DPP4, hACE2, or hDPP4. However, as our experimental data was limited this might restrict a comprehensive analysis of receptor usage in these viruses, such that it remains unclear which receptors the virus utilizes for cell entry.

The findings underscore the ongoing dissemination of CoVs among pika populations in the Qinghai-Tibet Plateau region and highlight the necessity for sustained research to assess the evolutionary patterns and potential public health risks associated with pika-borne CoVs.

## Supplementary Material

Fig S1_tree_partial_RdRp.pdf

Figures.zip

Fig S3_spike alignment.pdf

Supplementary tables.xlsx

Fig S4_tree_spike_aa.pdf

Fig S2_Genome comparison_A.pdf

## Data accessibility

Sequence reads have been deposited in the Short Read Archive BioProject PRJNA1128603. The complete or partial genome sequences of the CoVs generated in this study as well as the sequences of mitochondrial cytochrome *b* genes of the pikas have been submitted to GenBank and assigned accession numbers PP963831 – PP963865 and PP963933 – PP963967, respectively.
