# Supplementary figures and images for: Five-year longitudinal surveillance reveals the continual circulation of both alpha- and beta-coronaviruses in Plateau and Gansu pikas (Ochotona spp.) at Qinghai Lake, China1
Source: Emerg Microbes Infect. 2024 Aug 13;13(1):2392693. doi: 10.1080/22221751.2024.2392693 (PMC11346322; doi:10.1080/22221751.2024.2392693)

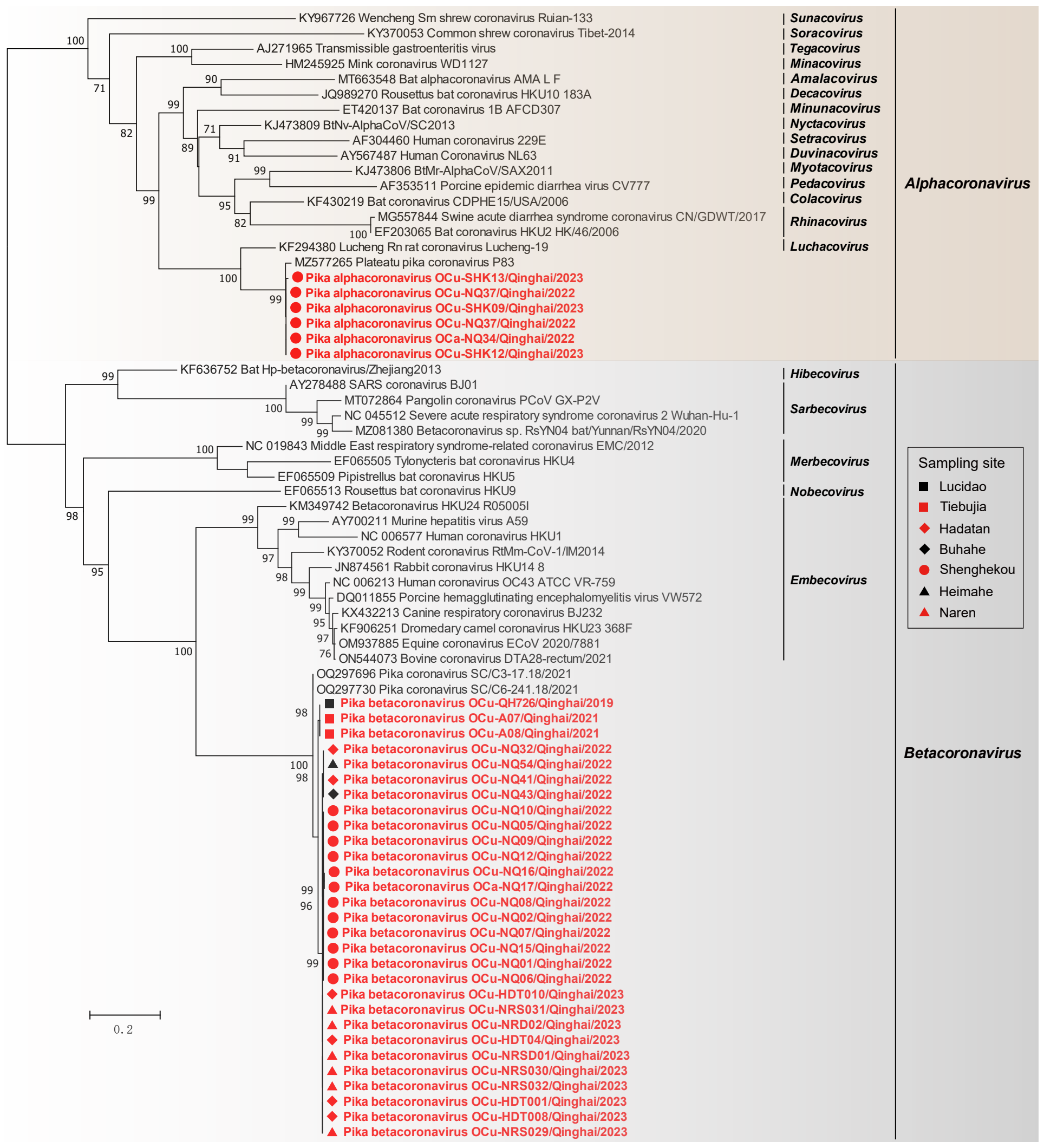

Supplement: Fig S1_tree_partial_RdRp.pdf [file TEMI_A_2392693_SM1612.pdf]

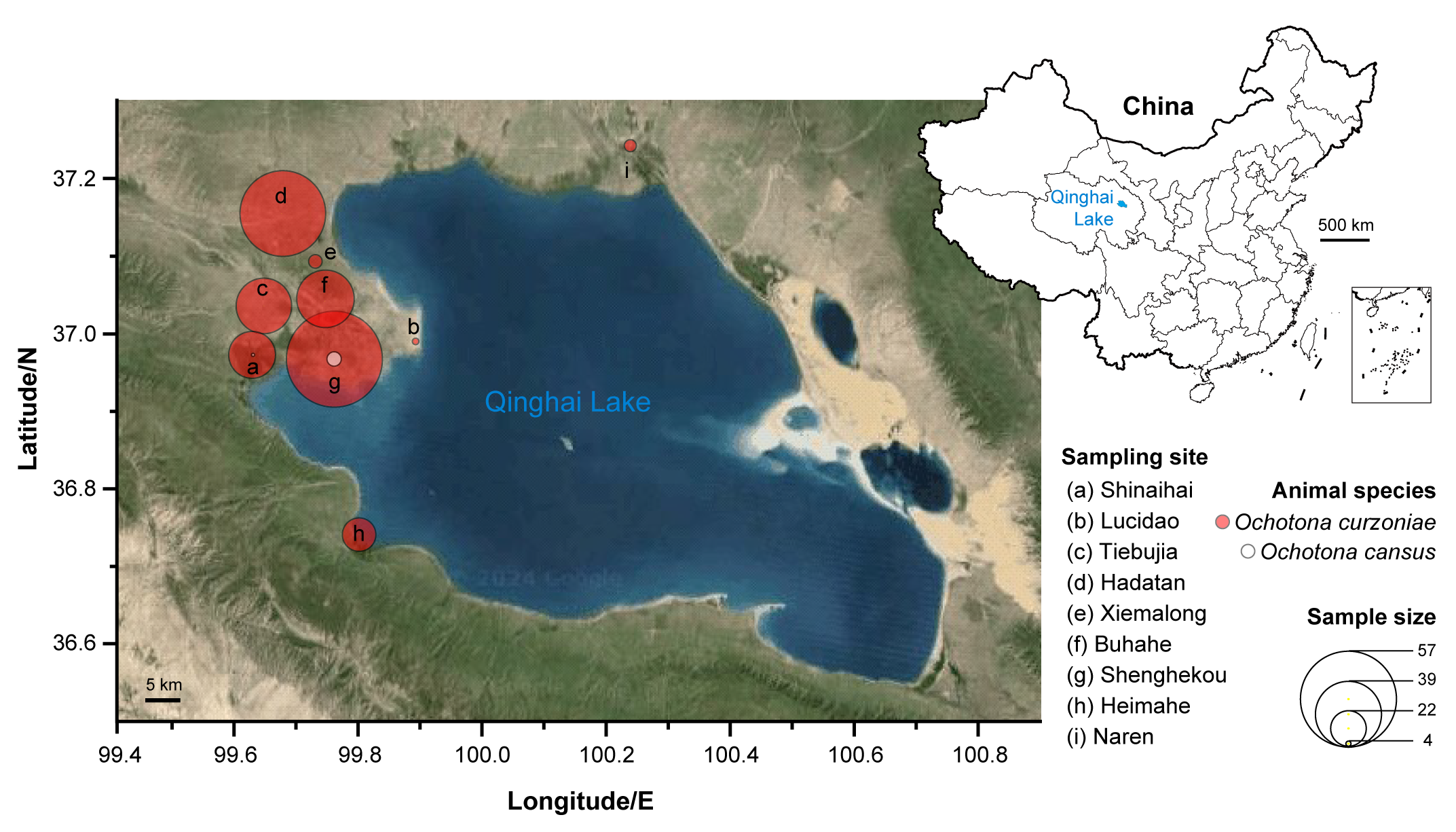

Supplement: Figures.zip [file TEMI_A_2392693_SM1611.zip › Fig 1_sampling.tif]

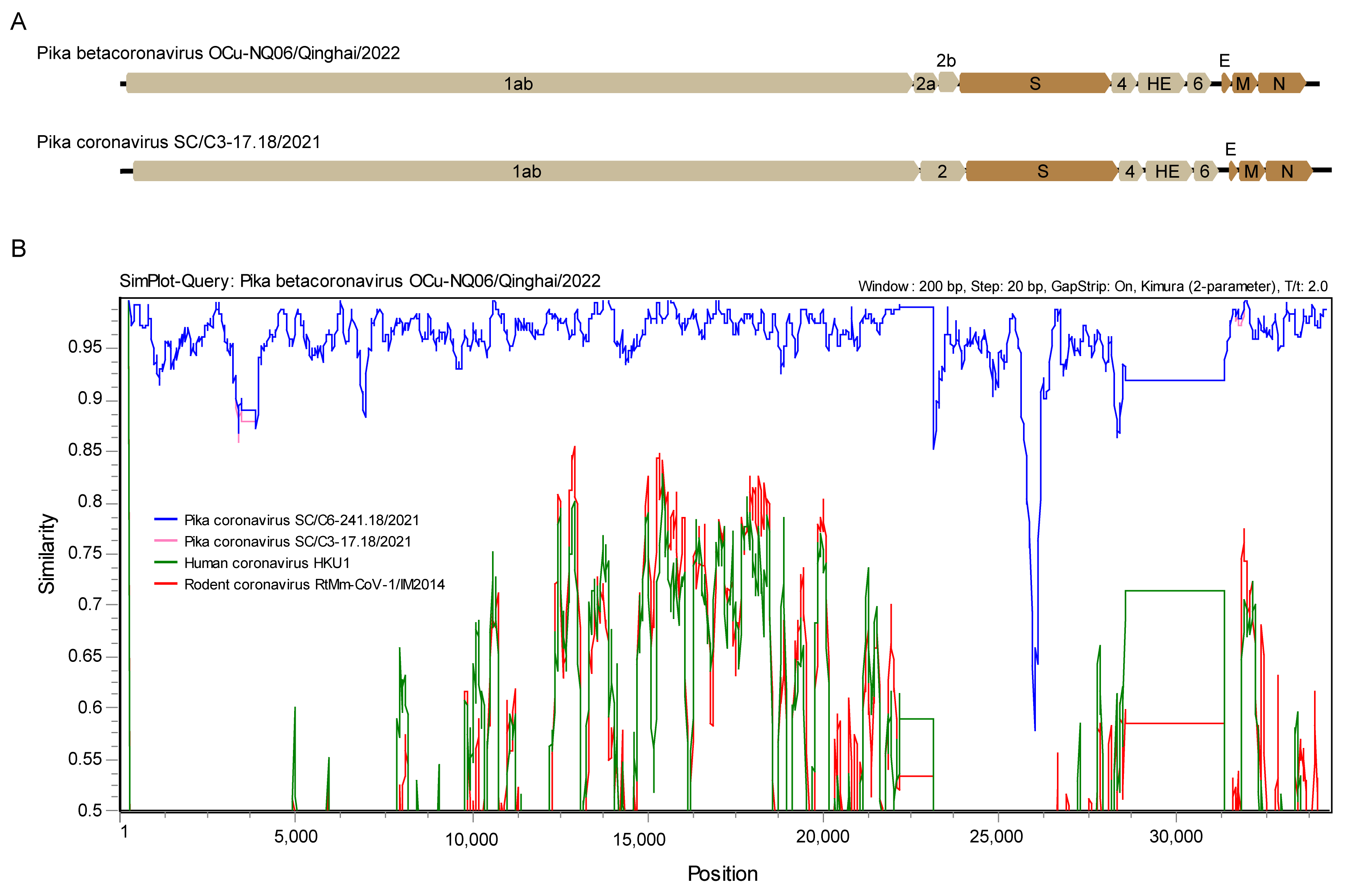

Supplement: Figures.zip [file TEMI_A_2392693_SM1611.zip › Fig 2_genome and recombination.tif]

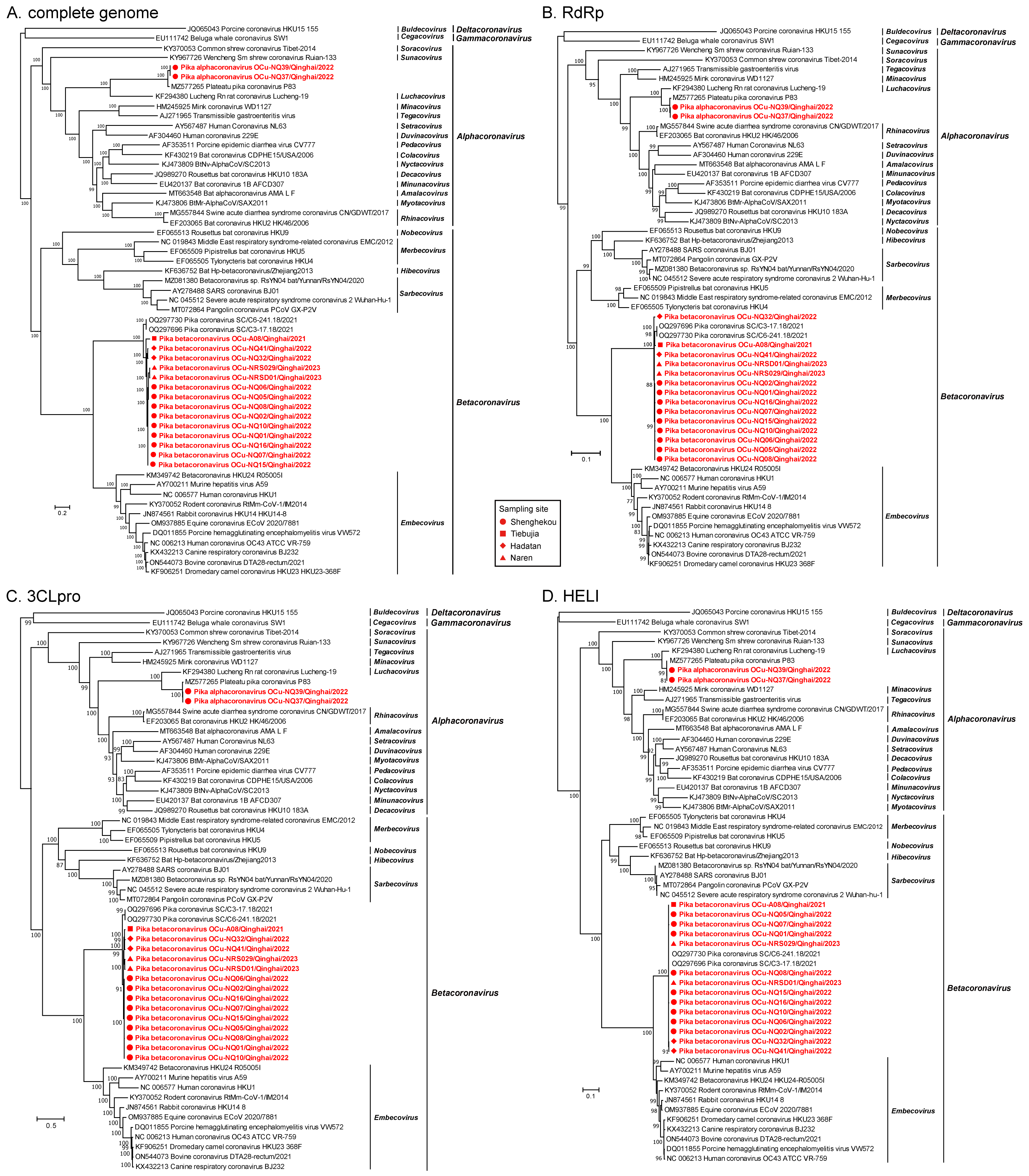

Supplement: Figures.zip [file TEMI_A_2392693_SM1611.zip › Fig 3_tree_4.tif]

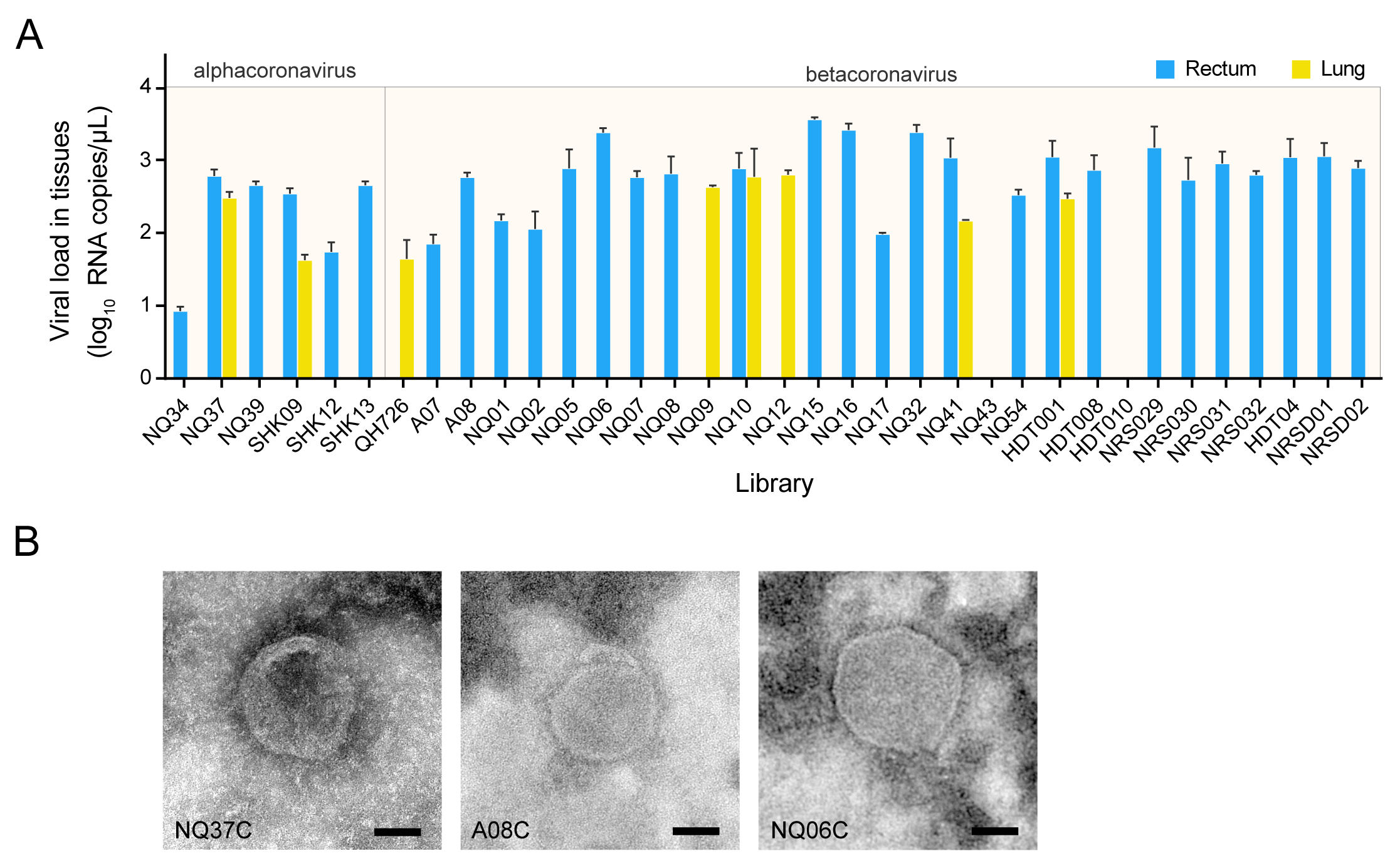

Supplement: Figures.zip [file TEMI_A_2392693_SM1611.zip › Fig 4_qPCR_TEM.tif]

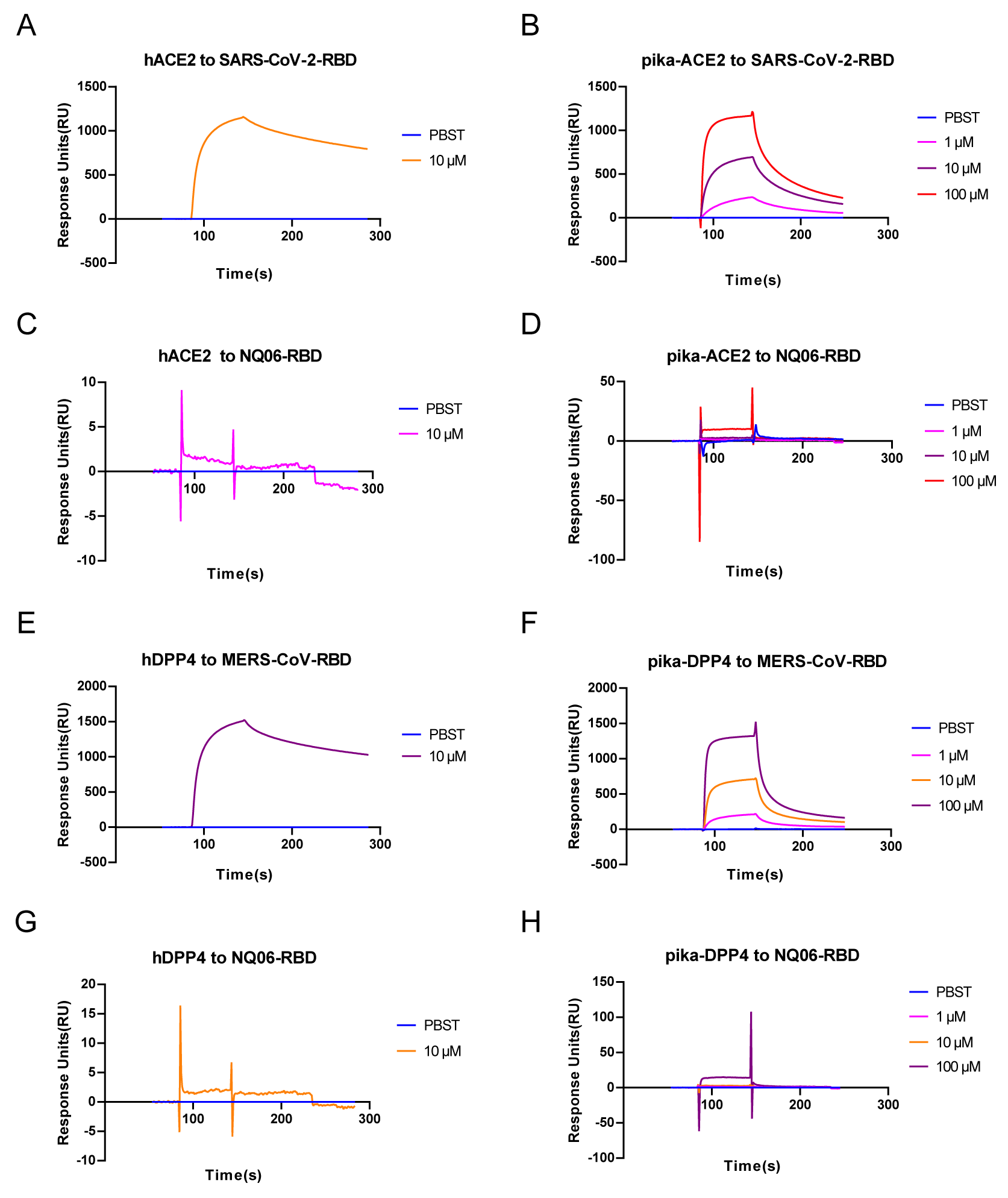

Supplement: Figures.zip [file TEMI_A_2392693_SM1611.zip › Fig 5_SPR.tif]

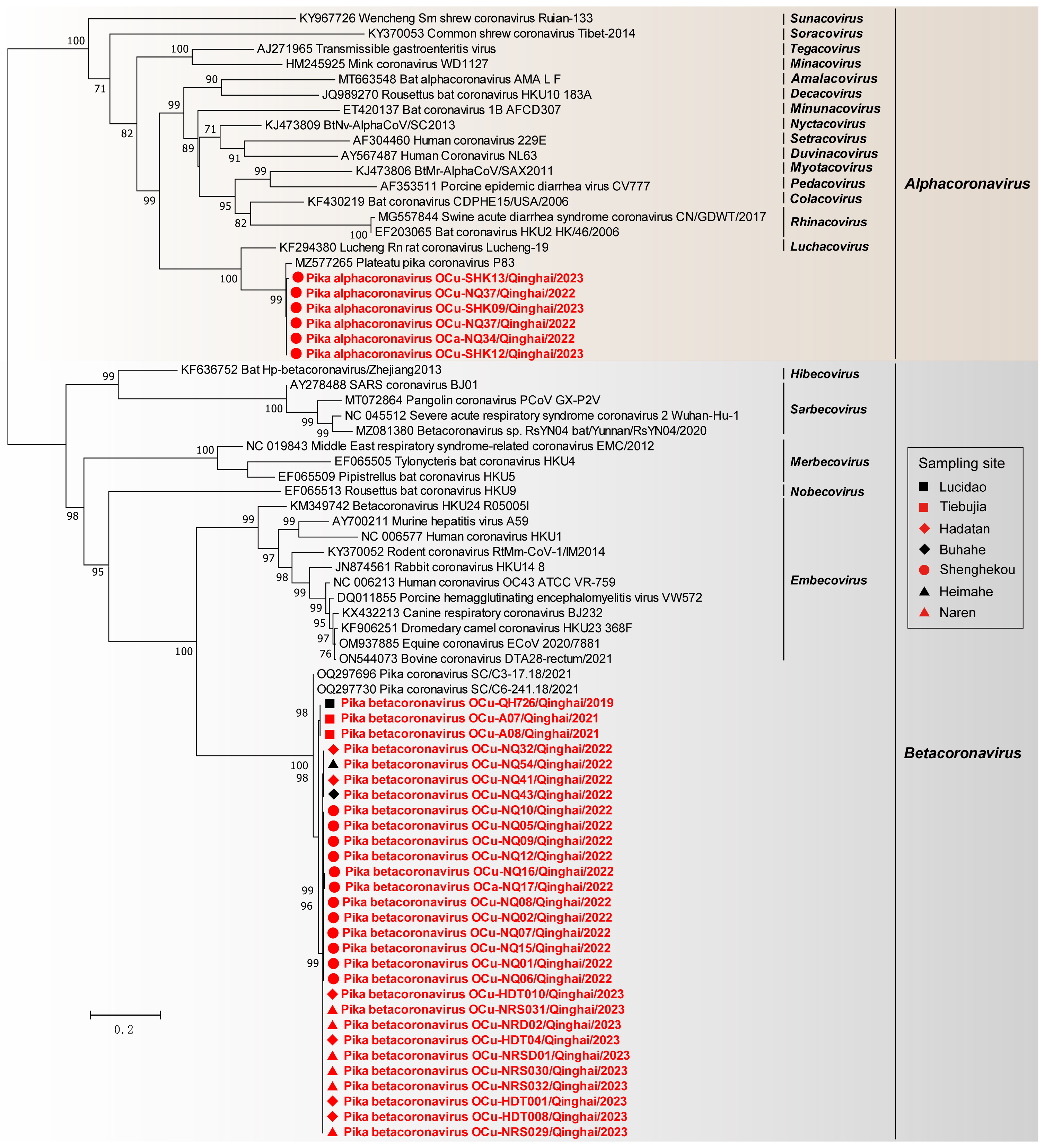

Supplement: Figures.zip [file TEMI_A_2392693_SM1611.zip › Fig S1_tree_partial_RdRp.tif]

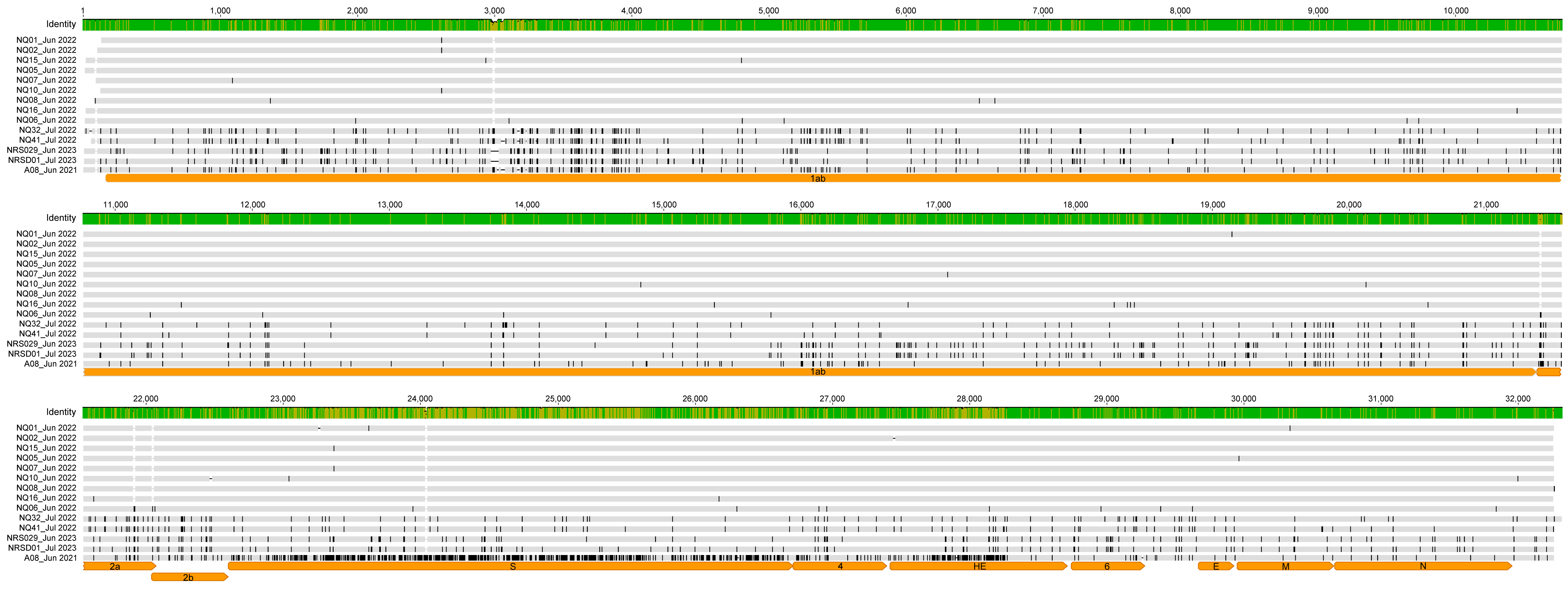

Supplement: Figures.zip [file TEMI_A_2392693_SM1611.zip › Fig S2_Genome comparison_A.tif]

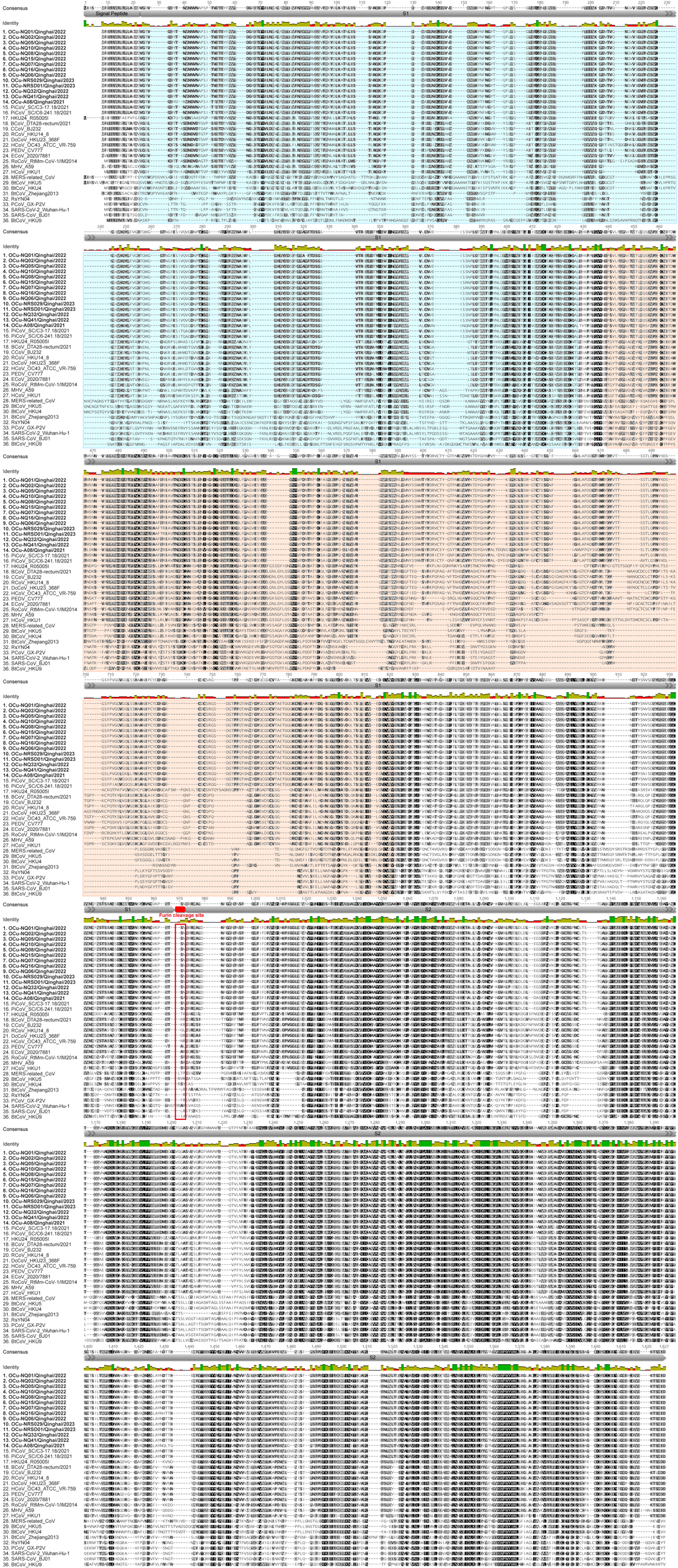

Supplement: Figures.zip [file TEMI_A_2392693_SM1611.zip › Fig S3_spike alignment.tif]

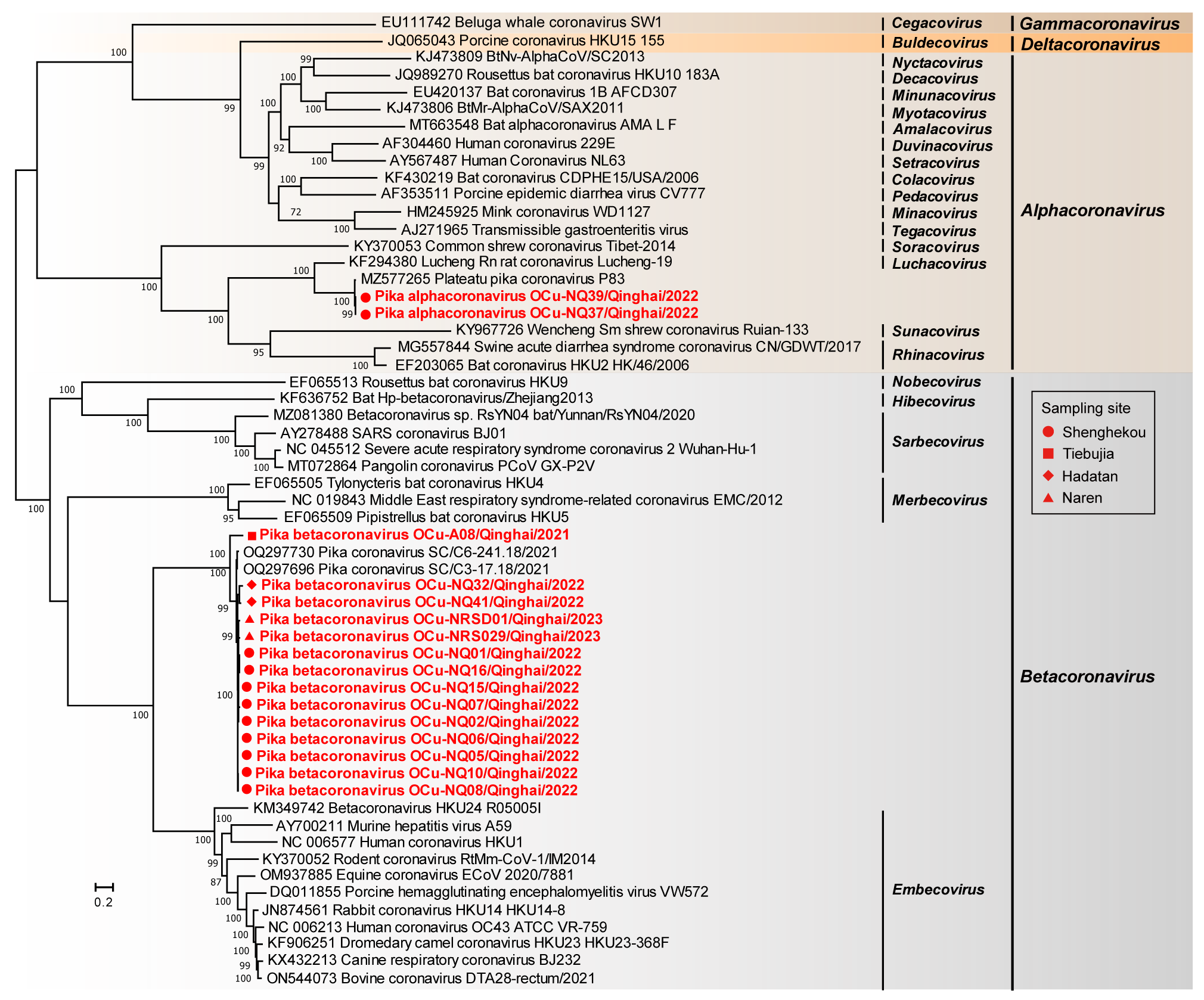

Supplement: Figures.zip [file TEMI_A_2392693_SM1611.zip › Fig S4_tree_spike_aa.tif]

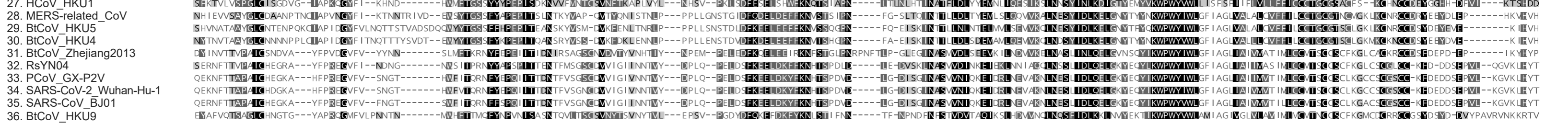

Supplement: Fig S3_spike alignment.pdf [file TEMI_A_2392693_SM1610.pdf]

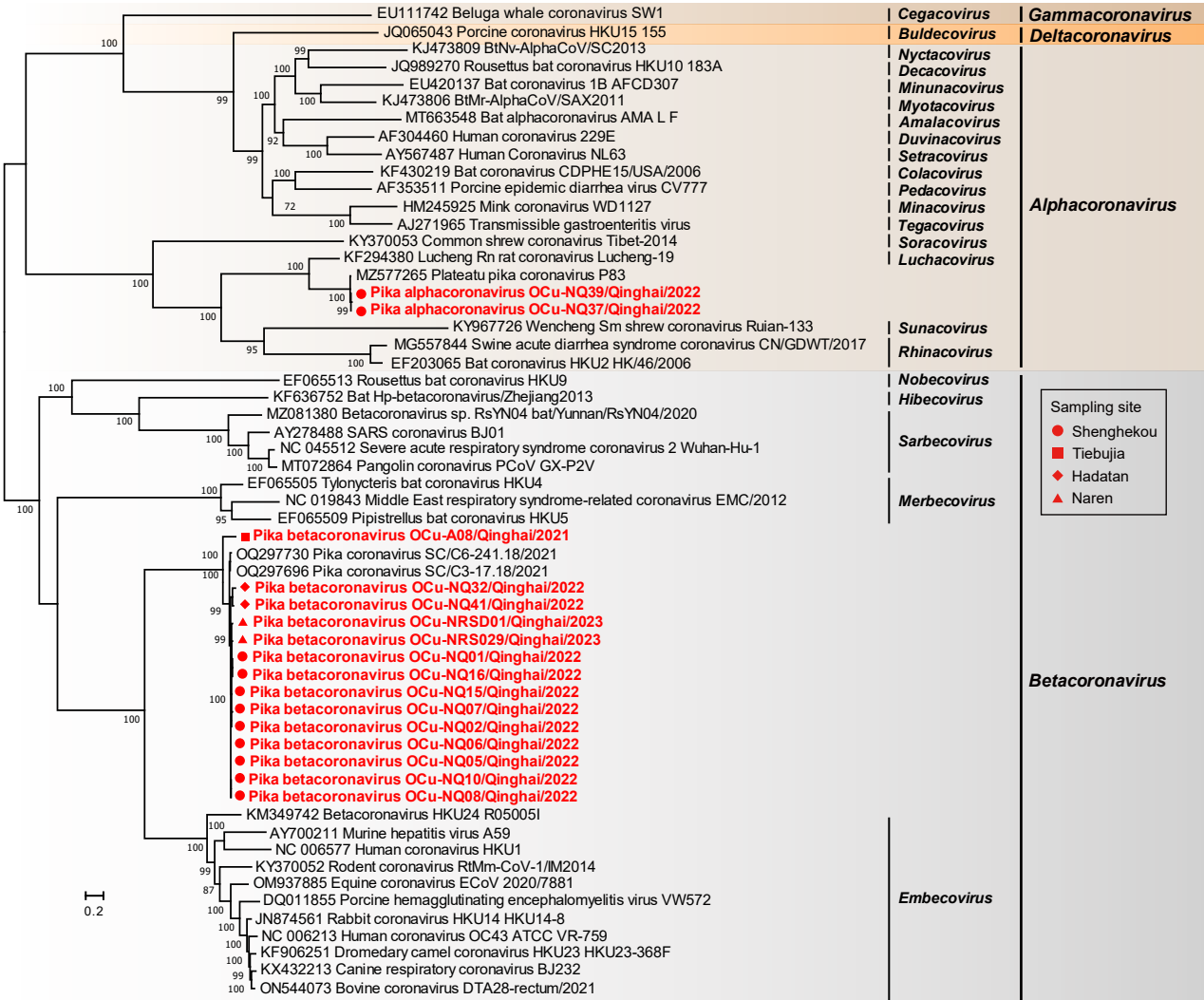

Supplement: Fig S4_tree_spike_aa.pdf [file TEMI_A_2392693_SM1608.pdf]

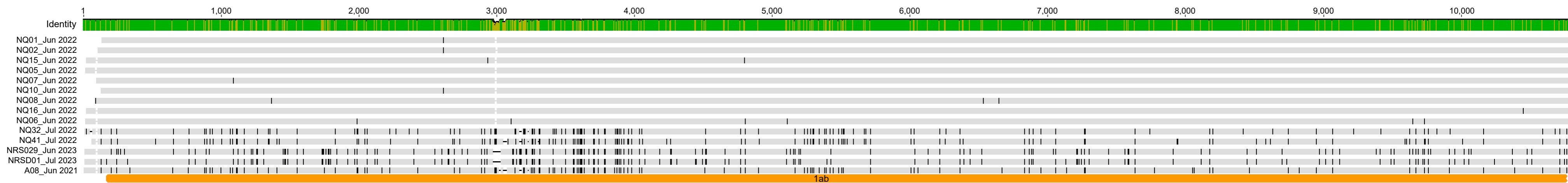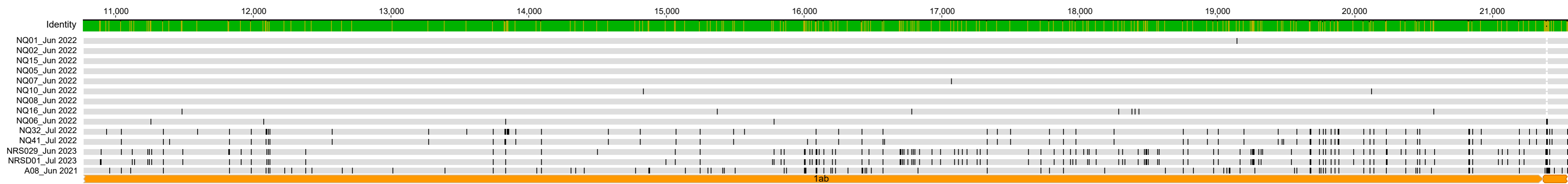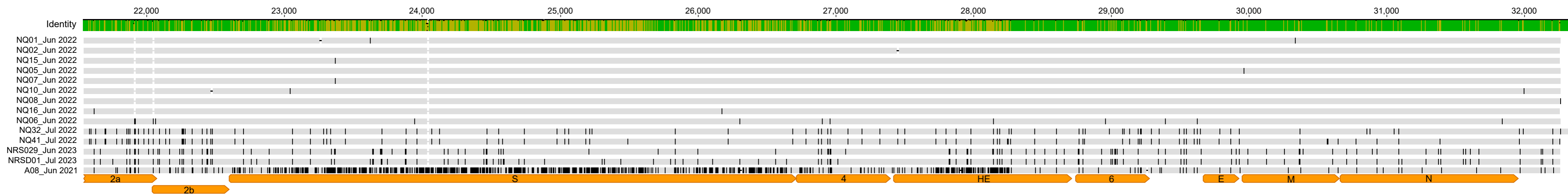

Supplement: Fig S2_Genome comparison_A.pdf [file TEMI_A_2392693_SM1607.pdf]
